# Relationships between stroke prevalence, health literacy, and oral health-related quality of life in middle-aged and older adults: a national survey study

**DOI:** 10.1186/s12877-023-03927-w

**Published:** 2023-04-18

**Authors:** Li-Chung Pien, Wan-Ju Cheng, Wen-Pei Chang, Su-Ru Chen, Kuei-Ru Chou, Chia-Hui Wang

**Affiliations:** 1 College of Nursing, Post-Baccalaureate Program in Nursing, Taipei Medical University, Taipei, 110301 Taiwan; 2 Psychiatric Research Center, Wan Fang Hospital, Taipei Medical University, Taipei, 116079 Taiwan; 3 Department of Nursing, Wan Fang Hospital, Taipei Medical University, Taipei, 116079 Chinese Taipei; 4 National Center for Geriatrics and Welfare Research, National Health Research Institutes, Miaoli, 350401 Taiwan; 5 Department of Public Health, China Medical University, Taichung, 406040 Taiwan; 6 Department of Psychiatry, China Medical University, Taichung, 404332 Taiwan; 7 Department of Nursing, Shuang Ho Hospital, Taipei Medical University, New Taipei City, 235041 Taiwan; 8 School of Nursing, College of Nursing, Taipei Medical University, No. 250, Wu-Xing Street, Taipei, 110301 Taiwan; 9 Research Center in Nursing Clinical Practice, Wan Fang Hospital, Taipei Medical University, Taipei, 116079 Taiwan; 10 Psychiatric Research Center, Taipei Medical University Hospital, Taipei, 110301 Taiwan; 11 Neuroscience Research Center, Taipei Medical University, Taipei, 110301 Taiwan

**Keywords:** Stroke, Health literacy, Oral health related quality of life, Older adults, National survey

## Abstract

**Background:**

Stroke may cause debilitating neurological deficiencies that result in motor, sensory, and cognitive deficits and poorer psychosocial functioning. Prior studies have provided some initial evidence for the significant roles of health literacy and poor oral health for old people. However, few studies have focused on the health literacy of individuals who had a stroke; therefore, the relationships between the health literacy and oral health-related quality of life (OHRQoL) among middle-aged and older adults who had a stroke are unknown. We aimed to assess the relationships between stroke prevalence, health literacy status, and OHRQoL in middle-aged and older adults.

**Methods:**

We retrieved the data from The Taiwan Longitudinal Study on Aging, a population-based survey. For each eligible subject, we gathered data in 2015 on age, sex, level of education, marital status, health literacy, the activity daily living (ADL), stroke history and OHRQoL. We evaluated the respondents’ health literacy by using a nine-item health literacy scale and categorized their health literacy level as low, medium, or high. OHRQoL was identified based on the Taiwan version of the Oral Health Impact Profile (OHIP-7T).

**Results:**

The final study contained 7702 community-based dwelling elderly people (3630 male and 4072 female) were analysis in our study. Stroke history was reported in 4.3% of participants, 25.3% reported low health literacy, and 41.9% had at least one ADL disability. Furthermore, 11.3% of participants had depression, 8.3% had cognitive impairment, and 3.4% had poor OHRQoL. Age, health literacy, ADL disability, stroke history, and depression status were significantly associated with poor OHRQoL after sex and marital status was adjusted. Medium (odds ratio [OR] = 1.784, 95% confidence interval [CI] = 1.177, 2.702) to low health literacy (OR = 2.496, 95% CI = 1.628, 3.828) was significantly associated with poor OHRQoL.

**Conclusions:**

Base our study results, people with stroke history had poor OHRQoL. Lower health literacy and ADL disability were associated with worse QHRQoL. Further studies are necessary to define practical strategies for reducing the risk of stroke and oral health with constantly lower health literacy, thereby improving the quality of life and providing health care of older people.

## Background

The 2019 Global Burden of Disease indicated that stroke is the second leading cause of death and the third leading cause of disability in the world [1]. The estimated global cost of stroke is over US$ 891 billion (1.12% of the global gross domestic product) [2]. The disease may cause debilitating neurological deficiencies that result in motor, sensory, and cognitive deficits and poorer psychosocial functioning. Researchers have identified an association between poor oral health and chronic systemic diseases such as ischemic stroke [3–5]. Despite advances in the discovery of modifiable and nonmodifiable risk factors and formulation of effective treatments, novel therapeutic approaches are urgently required to limit the growing burden of stroke [6].

Oral health–related quality of life (OHRQoL) is a multidimensional concept that implicates all aspects of daily activities [7]. Increasingly, OHRQoL has been used to evaluate oral treatment needs, oral health, and the consequences of dental treatment [7, 8]. A previous study observed that poor oral health status was directly associated with worse OHRQoL in Chilean older adults [9]. According to the World Health Organization, oral health will become an increasingly pressing issue as populations age around the globe [10]. Baniasadi et al. reported a positive association between low education level (eighth grade or lower), marital status, depression, smoking status, denture wearing, poor general health, tooth-induced pain, and periodontal diseases and poor OHRQoL among older people [11].

Oral diseases are common in patients with stroke, and stroke-related physical, sensory, and cognitive impairments can make oral health care challenging. Some examples of oral-health-care challenges in patients with stroke are stroke-associated orofacial motor deficits, decreased tongue pressure, and low-chewing efficiency, which in turn affects the ability to clear food debris out of the oral cavity and leads to poor oral hygiene. A study reported that patients with stroke had poorer clinical oral health status across various parameters (tooth loss, dental caries experience, and periodontal status) [12]. Zeng et al. found that patients who had stroke had poorer oral health than the healthy population, with more dental caries but fewer remaining teeth and worse periodontal status [13]. Periodontal disease is a risk factor for stroke, with the direct mechanism remaining unclear [14]. Health literacy involves obtaining, understanding, and using health information to make appropriate health decisions and follow treatment instructions [15]. Patients with inadequate health literacy report a poorer understanding of their medical condition [16], delayed diagnosis [17], low-self-management skills, lack of understanding of medical instructions, and low compliance with recommended treatments [18]. The level of health literacy is influenced by various determinants. The most frequently mentioned are education, age, and socio-economic factors [19]. An important argument for dealing with this issue are the results of various studies that prove problematic and completely inadequate health literacy in a relatively large part of the adult population [20, 21]. However, few studies have focused on the health literacy of individuals who had a stroke; therefore, reduced OHRQoL can be concluded for older people, with an unclear association to oral health and disease-related parameters. We aimed to assess the relationships between stroke prevalence, health literacy status, and OHRQoL in middle-aged and older adults.

## Methods

### The study

#### Aims

We aimed to assess the relationships between stroke prevalence, health literacy status, and OHRQoL in middle-aged and older adults.

#### Design

This study applied a cross-sectioned design.

### Study population and procedures

The study subjects were from the 8^th^ wave Taiwan Longitudinal Study on Aging (TLSA) [22]. The TLSA survey was a nationwide longitudinal study started in 1989, conducted by the institutes of the Health Promotion Administration of Taiwan. Every 3 to 4 years, the nationwide longitudinal study had consecutive follow-ups between 1989 and 2015, and the questionnaire collected by a group of trained interviewers [22, 23]. The TLSA survey applied a three-stage systematic random sampling design to select the equal probability of elderly samples from the townships, details of the sampling design and data collection methods can be found elsewhere [24–26]. In this study, subjects were from the TLSA survey in 2015, and the overall subjects were 3630 males and 4072 females, and the 8^th^ TLSA survey response rate was 70.7% [22].

### Measurements

#### Assessment of OHRQoL

We evaluated the self-reported OHRQoL using the Taiwan version of the Oral Health Impact Profile (OHIP-7T). The OHIP-7T scale was modified from the OHIP-14T [27], and it showed good psychometric properties and has been validated against OHIP-14T and self-reported dental symptoms [25]. The OHIP-7T comprised seven items, which indicate the frequency of an oral problem over the past 12 months, and responses are scored on a 5-point Likert scale ranging from 0 (*never*) to 4 (*almost*), with a maximum score of 28. Lower OHIP-7T scores indicated a higher OHRQoL [25, 26]. In Taiwan, the OHIP-7T was developed to assess the OHRQoL of older adults and was applied in the national TLSA survey from 2011 [22, 26]. In our study, good and poor OHRQoL were indicated by OHIP-7T scores of 0–14 and 15–28, respectively.

#### Stroke history assessment

We evaluated stroke history with a single binary-response question (“Do you have a history of physician-diagnosed stroke?”).

#### Health literacy assessment

The health literacy scale demonstrates good internal consistency reliability. Criterion-related validity was supported by its correlation with the Instrumental Activities of Daily Living and Life Satisfaction Index. Factor analysis indicated a three-factor structure. Known-group validity was supported by that people with good self-reported health status had better health literacy [28]. Responses were given on a 5-point Likert scale, and scores ranged from 9–45. A higher score indicated worse health literacy. The health literacy sum scores were ranked and divided into tertiles (ie, low, medium, and high).” It has been used in several studies and was found to be related to frailty and mental health [29–31].

#### Activities of daily living assessment

The activities of daily living (ADL) scale is a self-reported measure and was adapted to evaluate participant difficulties in performing the following six essential self-care activities of daily living: eating, bathing, dressing, showering, using the toilet, and getting in and out of bed or chair without assistance [32, 33]. Responses for each item were given on a 4-point Likert scale from 0 (*no difficulty*) to 3 (*completely impossible to do*), with a higher score indicating greater disability. The Cronbach’s α for the ADL scale was 0.87–0.94 [28, 32]. In the analysis, we divided ADL functioning into two levels, no disability and one or more disabilities.

#### Cognitive function assessment

We measured cognitive function using the 10-item Pfeiffer’s Short Portable Mental Status Questionnaire (SPMSQ) [34]. The SPMSQ questionnaire scored each item from 0 (*no error*) to 1 (*error*), with a higher score (total score: 0–10) indicating better cognitive function. A score of 8–10 indicated no cognitive function impairment, a score of 5–7 indicated mild cognitive impairment, a score of 3–5 indicated moderate cognitive impairment, and a score of 0–2 indicated severe cognitive impairment [34]. Participants were categorized into two subgroups: no cognitive impairment (SPMSQ = 0–2 errors) and cognitive impairment (SPMSQ ≥ 3 errors).

#### Assessment of depression

We used the total scale score from the Taiwanese version of the 10-item short-form Center for Epidemiological Studies Depression Scale (CESD-SF) to measure depression status [35]. Each item was scored from 0 (*rarely or none of the time*) to 3 (*all the time or* ≥ *4 days a week*), and the total score was distributed from 0–30. A higher score indicated more severe depression in the past week, and a CESD-SF score ≥ 10 indicated depression [35]; depression was coded as a binary variable (depression and no depression) in the analysis.

#### Social-demographic characteristics Assessment

There was a group of trained interviewers face-by-face to collect participants’ self-reported demographic characteristics, such as sex, age, education level, and marital status [22].

#### Ethical considerations

The Taiwan Medical University Ethics Committee (TMU-JIRB N201907030) approved our analysis of the data from the eighth wave of the TLSA survey.

#### Statistical analysis

All analyses were performed using SPSS 25.0 (IBM, Armonk, NY, USA). A *p* value < 0.05 indicated statistical significance. The categorical variables were summarized in terms of the frequency and percentage. We applied the Chi-square test for categorical variables to examine the differences between different stroke history groups. Multivariable logistic regression was applied to investigate the association between health literacy and stroke history and poor OHRQoL. Mental conditions (i.e., depression and cognitive function) and ADL were included as covariates. Other covariates included sex, age, and marital status. Because health literacy was highly correlated with education level, health literacy was removed from the multivariable logistic regression model.

## Results

Among the 7702 participants, 52.9% were women, 52.1% were aged between 50–64 years and the mean age was 63.32 years old (stand deviation was 9.60), and 74.1% of older people were married/cohabitating and 52.8% had an education level of high school or higher. Stroke history was reported in 4.3% of participants, 25.3% reported low health literacy, and 41.9% had at least one ADL disability. Furthermore, 11.3% of participants had depression, 8.3% had cognitive impairment, and 3.4% had poor OHRQoL, the OHRQoL mean scores was 3.12 stand deviation was 4.53 (Table 1).

**Table 1:** Distribution of demographic characteristics, health literacy, ADL, depression, cognitive function, and OHRQoL among participants (*N* = 7702)

Variable	Total	
	n/mean	%/SD
Gender		
Male	3630	47.1%
Female	4072	52.9%
Age (years)	63.32	9.60
50–64	4013	52.1%
65–74	2215	28.8%
75 and above	1474	19.1%
Education level		
Primary, can read	3632	47.2%
High school and above	4070	52.8%
Marital status		
Married/ Cohabitation	5704	74.1%
Single/ Widowed/ Divorced	1998	25.9%
Stroke history		
Yes	333	4.3%
NO	7369	95.7%
Health literacy	39.42	5.57
Low health literacy	1950	25.3%
Medium health literacy	2851	37.0%
High health literacy	2901	37.7%
ADL		
No disability	4476	58.1%
One disability and above	3226	41.9%
Depression	4.06	5.11
Yes	870	11.3%
No	6832	88.7%
Cognitive function	9.29	1.41
Cognitive impairment	640	8.3%
No cognitive impairment	7062	91.7%
OHRQoL	3.12	4.53
Good	7443	96.6%
Poor	259	3.4%

*ADL* activities of daily living, *OHRQoL* oral health–related quality of life

Table 2 displays the distribution of demographic characteristics, health literacy, ADL disability, depression, cognitive function, and OHRQoL stratified by stroke history. Among the participants with stroke history, 76.9% had at least one ADL disability, 22.8% had depression, and 21.9% had cognitive impairment, and they were more likely to have a poor OHRQoL than people without Stroke history. Participants with stroke history also had a higher rate of low health literacy (Table 2).

**Table 2:** Distribution of demographic characteristics, health literacy, ADL, depression, cognitive function, and OHRQoL stratified by stroke history (*N* = 7702)

Variable	Stroke history					
	No (*n*=7369)		Yes (*n* = 333)		X^2^	*P* value
Gender					31.56	< .001
Male	3423	46.5%	207	62.2%		
Female	3946	53.5%	126	37.8%		
Age (years)					102.36	< .001
50–64	3917	53.2%	96	28.8%		
65–74	2104	28.5%	111	33.3%		
75 and above	1348	18.3%	126	37.8%		
Education level					29.04	< .001
Primary, can read	3426	46.5%	205	61.6%		
High school and above	3943	53.5%	128	38.4%		
Marital status					0.25	0.617
Married/ Cohabitation	5462	74.1%	242	72.7%		
Single/ Widowed/ Divorced	1907	25.9%	91	27.3%		
Health literacy (score range 9–45)					55.92	< .001
High health literacy	2826	38.3%	75	22.5%		
Medium health literacy	2731	37.1%	120	36.0%		
Low health literacy	1812	24.6%	138	41.4%		
ADL					175.07	< .001
No disability	4399	59.7%	77	23.1%		
One disability and above	2970	40.3%	256	76.9%		
Depression					46.56	< .001
Had depression	794	10.8%	76	22.8%		
No depression	6575	89.2%	257	77.2%		
Cognitive function					84.37	< .001
Cognitive impairment	567	7.7%	73	21.9%		
No cognitive impairment	6802	92.3%	260	78.1%		
OHRQoL					64.30	< .001
Good	7147	97.0%	296	88.9%		
Poor	222	3.0%	37	11.1%		

Due to decimals, the total in some columns is less than 100%

*ADL* activities of daily living, *OHRQoL* oral health–related quality of life

Table 3 displays the multivariable logistic regression results. Age, health literacy, ADL disability, stroke history, and depression status were significantly associated with poor OHRQoL after sex and marital status was adjusted. Medium (odds ratio [OR] = 1.784, 95% confidence interval [CI] = 1.177, 2.702) to low health literacy (OR = 2.496, 95% CI = 1.628, 3.828) was significantly associated with poor OHRQoL. The presence of one or more ADL disabilities (OR = 2.712, 95% CI = 1.898, 3.875) was significantly associated with poor OHRQoL. People with a history of stroke (OR = 2.138, 95% CI = 1.442, 3.170) and depression (OR = 3.277, 95% CI = 2.482, 4.328) had a greater risk of poor OHRQoL. However, cognitive function impairment was not statically significantly associated with poor OHRQoL (Table 3).

**Table 3:** Adjusted OR in multivariable logistic regression models for poor oral health–related quality of life (*N* = 7702)

Variable	Poor OHRQoL		
	OR	(95% CI)	
Gender			
Male	1		
Female	0.959	0.727	1.267
Age (years)			
50–64	1		
65–74	1.509^**^	1.069	2.129
75 and above	1.676^**^	1.162	2.418
Marital status			
Married/ Cohabitation	1		
Single/ Widowed/ Divorced	1.307	0.990	1.725
Health literacy			
High health literacy	1		
Medium health literacy	1.784^**^	1.177	2.702
Low health literacy	2.496^***^	1.628	3.828
ADL			
No disability	1		
One disability and above	2.712^***^	1.898	3.875
Stroke history			
NO	1		
Yes	2.138^***^	1.442	3.170
Depression			
No depression	1		
Had depression	3.277^***^	2.482	4.328
Cognitive function impairment			
No cognitive impairment	1		
Cognitive impairment	1.154	0.813	1.640

Because education level and health literacy had high collinearity, education level was excluded from the logistic regression model

*ADL* activities of daily living, *OHRQoL* oral health–related quality of life

Statistically significant at

^*^for *p* < 0.05

^**^for *p* < 0.01

^***^for *p* < 0.001

## Discussion

The present study used a population-based cohort sample of Taiwan to explore the relationships between stroke prevalence, health literacy status, and OHRQoL in middle-aged and older adults. Health literacy is associated with better health status, healthier behavior, and better accessibility and use of healthcare facilities [19]. Health literacy can be improved by teaching skills or improving health services and may constitute a significant, modifiable determinant of self-care and health behavior [21]. The pooled prevalence of low health literacy ranged from of 27% to 48% in European Union Member States, depending on the literacy assessment method applied [36]. The prevalence rate of depression was above 7.5% among females aged 55–74 years and above 5.5% among males [37]. We reported 11.3% of participants had depression and 25.3% had low health literacy.

A stroke is an abrupt neurological outburst caused by impaired perfusion through the blood vessels to the brain. The stroke prevalence rates in the study population aged 60 years and older were 4.89% in the North China [38], 2.2% in Turkey [39], 1.4% in Indonesia [40]. We found the similar prevalence rate of stroke patients as the North China. The risk of stroke increases with age and doubles in men and women once they reach 55 years of age. Sex differences in stroke can arise at several key points in the disease that are dependent on age. Stroke risk is negatively correlated with age among women and positively but slightly correlated with age among men. This difference is likely to stem from many factors, such as social factors (i.e., decreased social support) and biological factors [41]. The development of depression after a stroke is the most frequent neuropsychiatric post-stroke complication. Meta-analyses have estimated the cross-sectional prevalence of depression after a stroke as between 18 and 33% [42]. The pathophysiology of depression after a stroke is complex and multifactorial and results from a combination of ischemia-induced neurobiological dysfunctions and psychosocial distress. Research has indicated neurobiological factors (rather than the psychological response to disability) as the main factors associated with depression after a stroke [43]. Health literacy is a modifiable risk factor for ischemic stroke [44]. Jeong and Cha reported that patients’ interest in health (*p* < 0.001), health literacy (*p* = 0.037), age (*p* = 0.001), and caregiver gender (*p* = 0.028) are significant factors influencing the health behavior of patients with stroke [45]. In our study, men had a higher percentage of stroke incidence than women. We also observed that older adults with a history of stroke had a higher rate of at least one ADL disability, low health literacy and depression.

Poor oral health can affect functional, psychological and social aspects of daily living, with a consequent impact on quality of life. Baniasadi et al. revealed a positive association between low education level (eighth grade or lower), marital status, depression, smoking status, denture wearing, poor general health, tooth-induced pain, and periodontal diseases and poor OHRQoL among older adults [11]. Al-Bitar demonstrated that periodontal diseases may negatively affect OHRQoL [46]. The patients with stroke have lower education level [47], poor oral hygiene practices [48] and buccal hemineglect [49] were the mechanisms found to significantly affect the OHRQoL. Low health literacy, at least one ADL disability, stroke history were the risk factors for poor OHRQoL in our study. Given the importance of oral health to overall health, health-care providers should conduct screening for oral health and mental health problems and effective management strategies should be devised and implemented. Therefore, medical staff and caregivers should be sensitized for oral health issues and support oral hygiene and dental (prevention oriented) consultations. In dental context, patients appear to need an interdisciplinary approach, addressing all important risks and needs of the patients, e.g., as displayed in the concept of individualized prevention [50, 51].

One strengths of this study are the use of a large national-representative sample of older adults. In addition, we included depression and cognitive functions in the statistical models because they are associated with oral health [52, 53]. Nevertheless, our study has several limitations. First, we excluded proxy variables from our analysis to strengthen the validity of the data; however, incorrect reporting may have happened. Generally, self-reported data are assumed to be reliable and accurate. However, the validity of self-reported data can vary and be influenced by several factors, such as the type of research tool, the wording of items, the respondents’ psychosocial conditions, and recall bias. Our study was a national survey study, and the sample was sufficiently large to avoid this bias. Second, although the multivariable analysis was performed to adjust for potential confounding factors, additional unmeasured confounding factors may have remained. Third, our findings may not generalize well to older adults outside Taiwan. Additionally, all the studied variables were cross-dependent, and more longitudinal analyses are required to evaluate further health literacy's effects on stroke prevalence and OHRQoL in the future. Finally, despite some psychometric properties of the OHIP-14T and OHIP-7T instruments having been evaluated in previous studies, we consider that there is a need for more robust statistical analyzes in order to generate better evidences about the validity of the factor structure of the instruments to the Taiwanese population using exploratory and confirmatory factor analysis. These analyses can contribute to generate better evidence of validity and reduce the chances of measurement bias.

## Conclusions

Base our study results, people with stroke history had poor OHRQoL. Lower health literacy and ADL disability were associated with worse QHRQoL. However, good OHRQoL is an integral part of overall health, but it is influenced by differences in oral health and the accessibility of healthcare services. Further studies are necessary to define practical strategies for reducing the risk of stroke and poor QHRQoL with constantly lower health literacy, thereby promoting health and quality of life for older people.

## Data Availability

The datasets used and analyzed during the current study are not publicity available but are available from the Ministry of Health and Welfare in Taiwan in https://www.hpa.gov.tw/EngPages/Detail.aspx?nodeid=1077&pid=6197 on request with the permission of the Ministry of Health and Welfare, Taiwan.

## References

[1] Collaborators GS (2021). Global, regional, and national burden of stroke and its risk factors, 1990–2019: a systematic analysis for the Global Burden of Disease Study 2019. Lancet Neurol.

[2] Owolabi MO, Thrift AG, Mahal A, Ishida M, Martins S, Johnson WD (2022). Primary stroke prevention worldwide: translating evidence into action. Lancet Public Health.

[3] Chang Y, Woo HG, Lee JS, Song TJ (2021). Better oral hygiene is associated with lower risk of stroke. J Periodontol.

[4] Dietrich T, Webb I, Stenhouse L, Pattni A, Ready D, Wanyonyi KL (2017). Evidence summary: the relationship between oral and cardiovascular disease. Br Dent J.

[5] Söder B, Meurman JH, Söder P (2015). Gingival inflammation associates with stroke-a role for oral health personnel in prevention: a database study. PLoS One.

[6] Hankey GJ (2017). Stroke. The Lancet.

[7] Gharehghani MAM, Bayani A, Bayat AH, Hemmat M, Karimy M, Ahounbar E (2021). Poor oral health-related quality of life among pregnant women: a systematic review and meta-analysis. Int J Dent Hyg.

[8] van de Rijt LJM, Stoop CC, Weijenberg RAF, de Vries R, Feast AR, Sampson EL (2020). The influence of oral health factors on the quality of life in older people: a systematic review. Gerontologist.

[9] Espinoza I, Thomson WM, Gamonal J, Arteaga O (2013). Disparities in aspects of oral-health-related quality of life among chilean adults. Commun Dent Oral Epidemiol.

[10] Listl S, Lavis JN, Cohen LK, Mathur MR (2022). Engaging citizens to improve service provision for oral health. Bull World Health Organ.

[11] Baniasadi K, Armoon B, Higgs P, Bayat AH, Mohammadi Gharehghani MA, Hemmat M (2021). The association of oral health status and socio-economic determinants with oral health-related quality of life among the elderly: a systematic review and meta-analysis. Int J Dent Hyg.

[12] Dai R, Lam OLT, Lo ECM, Li LSW, Wen Y, McGrath C (2015). A systematic review and meta-analysis of clinical, microbiological, and behavioural aspects of oral health among patients with stroke. J Dent.

[13] Zeng LN, Rao WW, Luo SH, Zhang QE, Hall BJ, Ungvari GS (2020). Oral health in patients with stroke: a meta-analysis of comparative studies. Top Stroke Rehabil.

[14] Guo T, Wang Y, Jiang Q (2022). Tooth loss and the incidence of ischemic stroke and transient ischemic attack: a systematic review and meta-analysis. J Healthc Eng.

[15] Institute of Medicine Committee on Health Literacy, Nielsen-Bohlman L, Panzer AM, Kindig DA, eds. Health literacy: a prescription to end confusion. Washington DC: National Academies Press. 2004.10.17226/10883. 25009856

[16] Aran N, Tregobov N, Kooij K, Harris D, Goel G, Poureslami I (2022). Health literacy and health outcomes in stroke management: a systematic review and evaluation of available measures. J Huma Soci Scie.

[17] Ravenell J, Leighton-Herrmann E, Abel-Bey A, DeSorbo A, Teresi J, Valdez L (2015). Tailored approaches to stroke health education (TASHE): study protocol for a randomized controlled trial. Trials.

[18] Wittink H, Oosterhaven J (2018). Patient education and health literacy. Musculoskeletal Science & Practice.

[19] Kickbusch I, Pelikan J M, Apfel F, Tsouros A. Health literacy. WHO Regional Office for Europe. 2013. https://apps.who.int/iris/handle/10665/326432.

[20] Kučera Z, Pelikan J, Šteflová A (2016). Zdravotní gramotnost obyvatel ČR výsledky komparativního reprezentativního šetření  [Health literacy in Czech population results of the comparative representative research]. Cas Lek Ces.

[21] Tiller D, Herzog B, Kluttig A, Haerting J (2015). Health literacy in an urban elderly East-German population - results from the population-based CARLA study. BMC Public Health.

[22] Taiwan Longitudinal Study on Aging (TLSA) [Internet]. 2022. Available from: https://www.hpa.gov.tw/EngPages/Detail.aspx?nodeid=1077&pid=6197.

[23] Sun CY, Yeh CY, Zhao Y, Chiu CJ (2020). Can individual attitudes toward aging rredict subsequent physical disabilities in older taiwanese individuals? a four-year retrospective cohort study. Int J Environ Res Public Health.

[24] Chang YH, Wu IC, Hsiung CA (2021). Reading activity prevents long-term decline in cognitive function in older people: evidence from a 14-year longitudinal study. Int Psychogeriatr.

[25] Kuo HC, Chen JH, Lai SK, Shen YC, Wang JC, Yang YH (2013). Development and validation of the taiwanese short-form of the Oral Health Impact Profile (OHIP-7T). Taiwan J Public Health.

[26] Tai CJ, Chen JH, Tseng TG, Lin Y, Hsiao YH, Lee MC (2020). Prediction of frailty and dementia using oral health impact profile from a population-based survey. Int J Environ Res and Public Health.

[27] Kuo HC, Chen JH, Wu JH, Chou TM, Yang YH (2011). Application of the Oral Health Impact Profile (OHIP) among Taiwanese elderly. Qual Life Res.

[28] Shih L, Hsieh CJ, Li PS, Liu CY (2021). Psychometric properties of the health literacy scale used in the Taiwan longitudinal study on middle-aged and older people. Healthcare.

[29] Shih YL, Hsieh CJ, Lin YT, Wang YZ, Liu CY (2021). The mediation effect of health literacy on social support with exchange and depression in community-dwelling middle-aged and older people in Taiwan. Healthcare.

[30] Tavousi M, Mohammadi S, Sadighi J, Zarei F, Kermani RM, Rostami R, Montazeri A (2022). Measuring health literacy: a systematic review and bibliometric analysis of instruments from 1993 to 2021. PloS One.

[31] Wang CH, Chang WP, Chen SR, Cheng WJ, Chou KR, Pien LC (2022). Health literacy and exercise to treat frailty in community-dwelling older adults: a national survey study. Int J Environ Res Public Health.

[32] Katz S, Ford AB, Moskowitz RW, Jackson BA, Jaffe MW (1963). Studies of illness in the aged: the index of ADL: a standardized measure of biological and psychosocial function. JAMA.

[33] Katz S, Downs TD, Cash HR, Grotz RC (1970). Progress in development of the Index of ADL. Gerontologist.

[34] Pfeiffer E (1975). A short portable mental status questionnaire for the assessment of organic brain deficit in elderly patients. J Am Geriatr Soc.

[35] Lee KL, Ou YL, Chen SH, Weng LJ (2009). The psychometric properties of a short form of the CES-D used in the Taiwan longitudinal study on aging. J Formosa Ment Health.

[36] Baccolini V, Rosso A, Di Paolo C (2021). What is the prevalence of low health literacy in European Union Member States? a systematic review and meta-analysis. J Gen Intern Med.

[37] World Health Organization, et al. Depression and other common mental disorders: global health estimates. World Health Organization, 2017. https://apps.who.int/iris/handle/10665/254610.

[38] Xia X, Yue W, Chao B, Li M, Cao L, Wang L (2019). Prevalence and risk factors of stroke in the elderly in Northern China: data from the national stroke screening survey. J Neurol.

[39] Köseoğlu Toksoy C, Bölük C, Türk Börü Ü, Akın S, Yılmaz AY, Coşkun Duman S (2018). Stroke prevalence in a coastal town on the Black Sea Coast in Turkey: community based study. Neurol Res Int.

[40] Setyopranoto I, Bayuangga HF, Panggabean AS, Alifaningdyah S, Lazuardi L, Dewi FST (2019). Prevalence of Stroke and associated risk factors in Sleman District of Yogyakarta Special Region. Indonesia. Stroke Res Treat.

[41] Gasbarrino K, Di Iorio D, Daskalopoulou SS (2022). Importance of sex and gender in ischaemic stroke and carotid atherosclerotic disease. Eur Heart J.

[42] Medeiros GC, Roy D, Kontos N, Beach SR (2020). Post-stroke depression: a 2020 updated review. Gen Hosp Psychiatry.

[43] Villa RF, Ferrari F, Moretti A (2018). Post-stroke depression: mechanisms and pharmacological treatment. Pharmacol Ther.

[44] Šedová L, Bártlová S, Hudáčková A, Havierniková L, Dolák F, Ostrý S (2021). Health literacy and modifiable risk factors of a stroke. Kontakt.

[45] Jeong J, Cha J (2020). Factors affecting health behavior of patients with stroke: focusing on health literacy of patients and family caregivers. Korean J Adult Nurs.

[46] Al-Bitar K. Oral health and quality of life: a clinic-based sample. PhD Thesis. Marquette University.2020.https://epublications.marquette.edu/theses_open/571.

[47] Leira Y, Seoane J, Blanco M (2017). Association between periodontitis and ischemic stroke: a systematic review and meta-analysis. Eur J Epidemiol.

[48] Dai R, Lam OLT, Lo ECM, Li LSW, Colman  M (2017). A randomized clinical trial of oral hygiene care programmes during stroke rehabilitation. J Dent.

[49] de Sire A, Baricich A, Ferrillo M, Migliario M, Cisari C, Invernizzi M (2020). Buccal hemineglect: is it useful to evaluate the differences between the two halves of the oral cavity for the multidisciplinary rehabilitative management of right brain stroke survivors? a cross-sectional study. Top Stroke Rehabil.

[50] Tew IM, Goo CL, Said SM, Zahari HI, Ali NA, Masawi FA (2020). Oral health related quality of life in stroke survivors at community-based rehabilitation centre: a pilot study. Makara J Health Res.

[51] Kwok C, McIntyre A, Janzen S, Mays R, Teasell R (2015). Oral care post stroke: a scoping review. J Oral Rehabil.

[52] Kisely S, Sawyer E, Siskind D, Lalloo R (2016). The oral health of people with anxiety and depressive disorders - a systematic review and meta-analysis. J Affet Disord.

[53] Lauritano D, Moreo G, Della Vella F, Di Stasio D, Carinci F, Lucchese A (2019). Oral health status and need for oral care in an aging population: a systematic review. Int J Environ Res Public Health.

